# Protective role of antifusarial eco-friendly agents (*Trichoderma* and salicylic acid) to improve resistance performance of tomato plants

**DOI:** 10.1016/j.sjbs.2022.01.020

**Published:** 2022-01-15

**Authors:** Ameena A. AL-surhanee

**Affiliations:** Biology Department, College of Science, Jouf University, Sakaka 2014, Saudi Arabia

**Keywords:** *Trichoderma harzianum*, Metabolic, Immune system, Isozymes, Oxidative stress traits, Tomato plants

## Abstract

*Fusarium* wilt triggered great losing in tomato plants quality and quantity in all worlds. In the recent experiment, physiological resistance performance in tomato seedlings using *Trichoderma harzianum* and salicylic acid (SA) either (individual or combination) anti *Fusarium* had been studied. *In vitro* antifungal prospective of *T. harzianum* and SA against F. oxysporum were also examined. A noticeable antifungal capacity with highest activity of 10 and 8 mm ZOI after the treatment with the *T. harzianum* and SA*.* Also, *Trichoderma* have great ability to decreasing *Fusarium* growth by 25% inhibition at dual culture method. The MIC of SA was 1.5 mM to reduce *Fusarium* growth. For more ultrastructure by TEM of *Fusarium* treated with SA and *Trichoderma* showed alteration of cell wall as well as cytoplasmic components of mycelium, macroconidia and microconida. In the current experiment, ameliorative potentials of *T. harzianum* and SA either (individual or combination) via soil or foliar application were administered to the *Fusarium*- infected tomato plants and then disease index, growth indicators, photosynthetic pigments, metabolic markers, and antioxidant isozymes were assessed. The achieved result indicates that *T. harzianum* and SA through two modes (foliar and soil) lowered PDI by 12.50 and 20.83% and produced great protecting ability by 86.36 and 72.2%. The results revealed, infected seedlings exhibited high decrement in all tested growth characters, photosynthetic pigment contents, contents of total carbohydrate and protein, whereas proline, phenols and enzymes’ activity were elevated under *Fusarium* infectivity. It was concluded that use of combination (*T. harzianum* and SA) acted as a commercially eco-friendly instrument for intensifying the defense system of tomato plants against *Fusarium* wilt.

## 1 Introduction

Under today’s era of increased globalized climatic and natural disturbances, one of the principal daunting challenges for farmers is to produce food and other resources for the burgeoning world population, that has been estimated to accelerate at a rate of about 1.05 % per year (Loudiere and Gourbesville, 2020). Researchers working in the current field proposed that to cope with these pressures by 2050, it is indispensable to maximize the production of important food crops by 87% (Fróna et al., 2019).

Wilt disease caused by *F. oxysporum* destructively affects plants, as significantly decreasing the crop (Abada and Eid, 2014). It should take into account that from the most dangerous nurse fungus, which negatively affect the production of many plants, such as such as tomatoes, peppers, egg plant, and watermelons and therefore hit the agricultural economy (Bebber and Gurr, 2015, Igiehon and Babalola, 2017, Ortega et al., 2020). Fungal diseases trigger the increase of reactive oxygen species (ROS) such as superoxide radicals, singlet oxygen and hydrogen peroxide (H_2_O_2_) that limits many important physiological processes, absorption and transport of water and nutrients, as well metabolic products(Aldinary et al., 2021), which harm plant growth. During evolution, plants have established various adaptive strategies to withstand under fungal attack as development of growth and metabolic adaptations (Bai et al., 2018, Zhao et al., 2018).

The impact of ecological resources as valuable strategies to lessen the effect of fungal disease so as to enhance the yield and ability of plants to survive under stress were evaluated. Eco-friendly agents carrying therapeutic nutrition materials are aimed at supplying plants with nutrients, vitamins and hormones which are responsible for growth in addition to inhibiting pathogen attach directly or indirectly (Aldinary et al., 2021, Kumar and Aloke, 2020). The physiological immunity is the case of activating the compounds responsible for defence within the plant (Enyedi et al., 1992). The species of *Trichoderma* are considered rhizosphere colonizing fungi, non-pathogenic and has super capacity to inhibit the work of pathogens and at the same time as motivation for plant growth, by producing many antioxidants that protect the plants from the burst conditions that are exposed to the roots due to injury, this is next to the competition with nutrition and production of aromatic compounds as a means of defence of plants, also produce analytical enzymes of the cellular pathogens in soil (Perello et al., 2016). Salicylic acid (SA) is natural derivative and performs an important role in the transfer of defensive signals within cells and is an expressive material for systemic resistance (Mandal et al., 2009). SA performs a vital role and stimulates growth and increases the efficiency of the plant absorption and to carry out the photosynthetic process, which positively affects the anatomical structures of plant leaf, stimulating vegetative growth and resistance against pathogen attack (Emamverdian et al., 2020). This study was aimed for deeply understanding of the ability of *T. harzianum* and SA in inhibiting the growth traits of *F. oxysporum f. sp. Lycopersici* and the possibility of improvement and revitalizing the signals responsible for defence within the tomato plant against the disease of wilt to minimize the use of fungal pesticides that have proven severely damage to public health and the environment.

## 2 Materials and methods

### 2.1 Application methods and source of inducers

*T. harzianum* were collected from Al-Azhar Center for Fermentation Biotechnology and Applied Microbiology (Ferm-bam) Al-Azhar University, Nasr City, Cairo, Egypt and were maintained on slants of PDA and stored at 4 °C till further use each treatment using 2 ml/one plants. The preparation of *F. oxysporum f. sp. Lycopersici* (pathogen) inoculums was accomplished by following (Aldinary et al., 2021) method.

### 2.2 In-vitro antifungal activity

The antifusarial activity of SA and *Trichoderma* was established as the technique explained by(He and Wolyn, 2005), MIC of SA was defined by SA concentrations (0. 5, 1, 1.5, 2 and 2.5) mM.

### 2.3 Dual culture

According to(Chen et al., 2021), *T. harzianum* was placed 9 cm apart on the opposite side of *Fusarium* on PDA plate supplemented with 2 g/l chloramphenicol at 30 °C for 5 days with three replicates, aggressive activity was documented by the following formula: % PI = [(r1 - r2)/r1] × 100, where r1 is the distance between the end point and cultural point of the *F. oxysporum* where r2 represents the distance between the sowing point and the edge of the *F. oxysporum* from *T. harzianum*.

### 2.4 Ultra-structure

The cytological variations generated in *Fusarium* treated with *T. harzianum* and SA were examined with a JOEL JM 100-C electron microscope. The samples were handled and post fixed according to (Lin and Langenberg, 1983).

## 3 Experimental setup

Four weeks old seedlings of tomato (*Solanum lycopersicum* L. *var*. 023) were achieved from Ministry of Agriculture Al Jouf, Saudi Arabia. Uniform looking seedlings were sown in pots (40 × 40 cm) having mixture of 7 kg sand and clay (1:3), in a plastic greenhouse. The pots were placed in the greenhouse maintained at 22/18 °C day/night T and 70–85% relative humidity. The plants were irrigated normally with tap water for five days. *T. harzianum* and SA were treated for 7 days after injection with *F. oxysporum f. sp. Lycopersici*. A complete block design experiment and two controls each consisting of six replicates was used. Each pot contained one plant. The six treatments were; I-healthy control; 2-infected control; 3-infected + *T. harzianum* (through soil); 4-infected *+ T. harzianum* (through foliar); 5-infected *+* SA (through soil); 6- infected + SA (through foliar); 7-infected + (*T. harzianum* + SA) (through soil); 8-infected + (*T. harzianum* + SA) (through foliar).

### 3.1 Disease symptoms and disease index

The disease symptoms were assessed on 60 days old plants and the disease index and % of inducers protection were evaluated according to (Farrag et al., 2017).

### 3.2 Vegetative growth and metabolic parameters as resistance indications

Growth parameters including shoot length (cm), root length (cm) and number of leaves per plant were noted after harvesting the samples.

Assessment of chlorophyll (a and b) and carotenoid were determined according to (Goedheer et al., 1966, Lichtenthaler and Buschmann, 2001) methods.

The total phenolic content was estimated by following method. The content of soluble protein was assessed according to (Kashyap et al., 1980). The method of (Bates et al., 1973) was used for estimation of proline. The soluble sugar content was assessed by anthrone based method and absorbance of reaction mixture was measured at 625 nm (Irigoyen et al., 1992).

The Bergmeyer, method was employed for peroxidise (POD) activity determination. The polyphenol oxidase (PPO) activity was analysed by the (Lavid et al., 2001) protocol.

### 3.3 Statistical analysis

The results are the means ± standard error (n = 3). The analysis of variance (ANOVA) and Tukey's HSD test was used to determine the significance level at *p ≤ 0.05* by using Minitab 17.

## 4 Results

### 4.1 *In vitro* studies

A - Dual culture

**Fig. 1:**
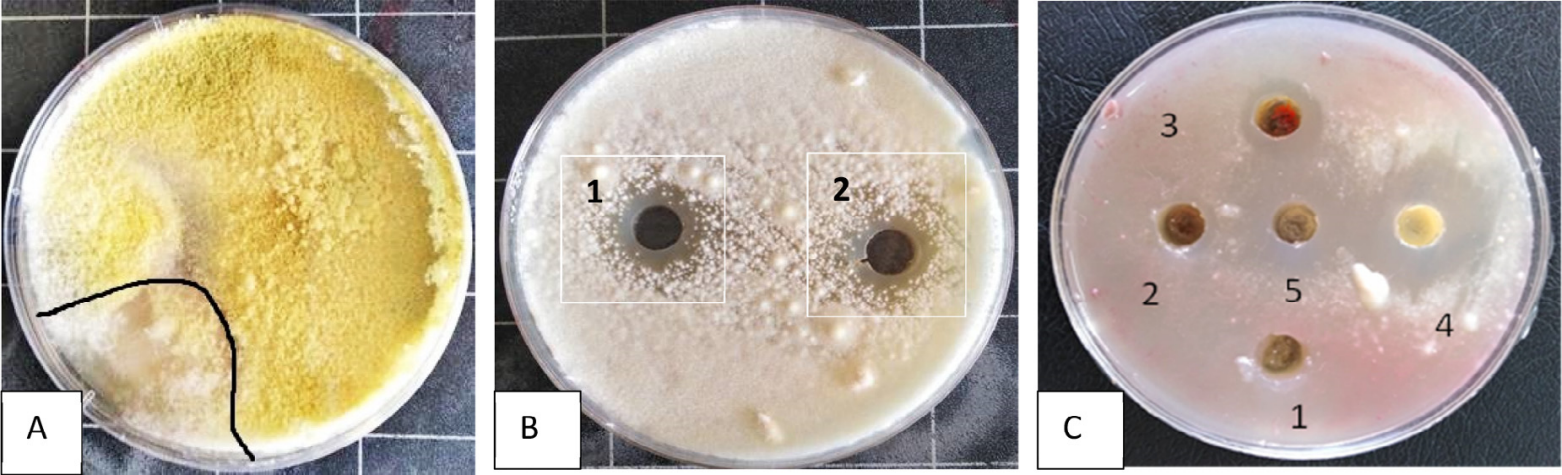
A: dual culture, B: antifungal activity of 1: *Trichoderma*, 2: salicylic acid and C: MIC of salicylic acid on *Fusarium oxysporum*.

Results in Fig. 1 indicated that the percentage of inhibition was 25% of *F. oxysporum* by *T. harzianum,* where *Fusarium* singly 2 cm singly but under treatment of *Trichoderma* 1.5 cm.

B - Antifungal activity of *Trichoderma* and SA on *Fusarium*

Results in figure (1 A) showed that antifungal activity of *Trichoderma* and SA against *F. oxysporum* in vitro, where *Trichoderma* highly showed inhibition zone (10 mm diameter). Also, its SA recorded antifungal activity at MIC 1.5 Mm by 8 mm inhibition zone diameter figure (1B).

C - Ultra-structure responses

**Fig. 2:**
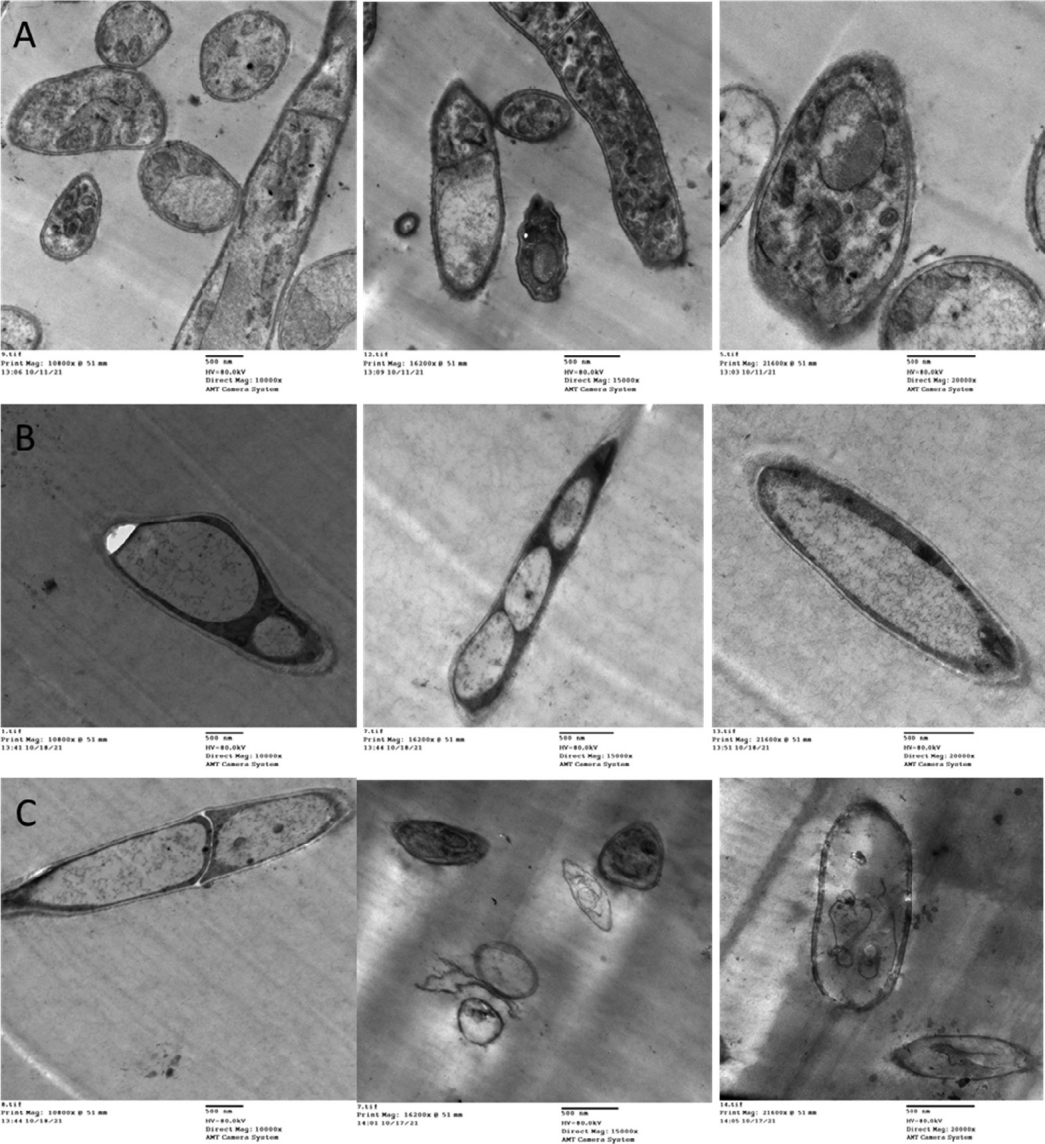
Ultra-structure of *Fusarium oxysporum* under controlling by *Trichoderma* and salicylic acid: where A: represent control *Fusarium*, B: effect of salicylic acid and C: effect of *Trichoderma* on *Fusarium oxysporum*.

Results in Fig. 2 showed that ultra-structure features of mycelium, macroconidia and microconida of *F. oxysporum* were affected by treatment with SA and *Trichoderma*. There are noticed changes in morphological cell wall and membranes as well as cytoplasmic contents were not distinguished compared with control. Whereas, SA caused deformation of mycelium, macroconidia and microconida of *Fusarium* through deposition of cytoplasmic components on cell wall compared with control. But *Trichoderma* caused moderate destroyed of *Fusarium* structure through elongated of macroconidia with irregular cell wall and compact and or losing cytoplasmic component compared with control.

### 4.2 In vivo studies

A - Percent disease incidence (PDI) and percent protection (P%)

Table 1 revealed that tested inducer application either (individual or combination) significantly minimized *F. oxysporum* induced wilt PDI in comparison with control plants. Conversely, the data indicated that, the infection percent reached 91.66% in infected control plants. Combination of *Trichoderma* and SA (through foliar and soil) was the best application method and decreased the PDI by 12.5 and 20.83% and resulted in high defense by 86.36 and 72.2%. The treatment by *Trichoderma* (through soil) reduced PDI by 29.16% and caused high protection by 68.18%, and came next treatment with SA (through soil and foliar) that reduced PDI by (33.33 and 37.5%) and caused protection by (59.08 and 63.63%)**.**

**Table 1:** Effect of *Trichoderma* and Salicylic acid on disease index of infected Tomato plants:

Treatments	Method of application	Disease symptoms Classes					DI (disease index) (%)	Protection (%)
				
Tri	through soil	4	0	0	1	1	29.16	68.18
SA		2	2	1	0	1	33.33	63.63
Tri + SA		4	0	1	1	0	20.83	77.2
Tri	through Foliar	1	0	2	1	2	62.5	31.81
SA		3	0	1	1	1	37.5	59.08
Tri + SA		5	0	0	1	0	12.5	86.3
Control infected		0	0	0	2	4	91.66	0

B - Growth biomarkers

As evident from Table 2, the morphological biomarkers (plant height and number of leaves) were affected by tested inducers at either method application (soil & foliar). It is clear from the Table 2 that *F. oxysporum* induced negative impacts on all tested vegetative traits. In comparison with healthy untreated control, *F. oxysporum* infected plants had a depressive effect on shoot length by 41.50%, root length by 43.89% and leaf number by 63.65%. Concerning, the effect of tested inducers on infected plants, it was noticed that all morphological biomarkers were significantly improved at both method application (soil & foliar), whereas, the best treatment was Tri + SA through foliar and soil, respectively versus infected control plants**.**

**Table 2:** morphological indicators of tomato plant treated with *Trichoderma* and Salicylic acid:

Treatments	Method of application	Shoot length(cm)	Root length(cm)	Number of leaves per plant
Tri	through soil	47.25 ± 1.75 ^bc^	21.06 ± 0.89^b^	12.44 ± 0.42^b^
SA		**34.12 ± 1.2 ^d^**	**15.04 ± 0.87 ^d^**	**7.97 ± 0.22 ^cd^**
Tri + SA		50.16 ± 1.33 ^ab^	21.9 ± 0.33^b^	13.16 ± 0.25^b^
Tri	through Foliar	**47.11 ± 1.6 ^bc^**	**16.38 ± 0.32^c^**	**9.97 ± 0.41^c^**
SA		46.37 ± 1.75^c^	16.09 ± 1.18 ^e^	9.47 ± 3.7^c^
Tri + SA		**50.72 ± 1.16 ^a^**	**21.33 ± 0.32^b^**	**16.38 ± 0.12 ^a^**
Control infected		30.18 ± 0.86 ^e^	13.14 ± 0.05^f^	6.52 ± 0.55 ^d^
Control healthy		**51.62 ± 0.87 ^a^**	**23.42 ± 1.14 ^a^**	**17.94 ± 0.45 ^a^**
LSD at 0.05		2.495	1.314	2.365

C - photosynthetic pigments

The photosynthetic pigments (Chl a and Chl b) exhibited a decline in plants infected with *F. oxysporum* (Table 3). Out of three photosynthetic traits, carotene contents showed non-significant increase in comparison to control healthy plants. It was found that clear positive responses in photosyntheticpigments (Chl a and Chl b) through application of elicitors. These effects varied considerably with the mode (foliar or soil) of usage. However, when infested plants treated with (Tri + SA, SA and Tri) through soil were the best treatments that showed a marked improve in chlorophyll *a* & b, followed by (Tri + SA, SA and Tri) through foliar respectively as compared to control (Table 3). Also, the found results proved that in *Fusarium*-infected plants, carotenoids contents were improved in response to the treatment with (Tri + SA, Tri and SA) through foliar and soil application, respectively**.**

**Table 3:** Photosynthetic pigments (Ch a, Ch b and carotenoid) of tomato plant treated with *Trichoderma* and Salicylic acid through (soil and foliar) application.

Treatments	Method of application	Chlorophyll *a* (mg/ g fresh weight)	Chlorophyll *b* (mg / g fresh weight)	Carotenoid (mg/ g fresh weight)
Tri	through soil	6.3 ± 0.03^b^	4.88 ± 0.08^b^	1.24 ± 0.08^b^
SA		6.74 ± 0.25 ^a^	7.81 ± 0.43 ^a^	0.93 ± 0.05^c^
Tri + SA		6.95 ± 0.02 ^a^	7.89 ± 0.16 ^a^	1.84 ± 0.01 ^a^
Tri	through Foliar	4.46 ± 0.028 ^de^	3.24 ± 0.02^d^	0.42 ± 0.15 ^ef^
SA		4.72 ± 0.12 ^d^	3.78 ± 0.10^c^	0.71 ± 0.01 ^d^
Tri + SA		5.22 ± 0.215^c^	4.01 ± 0.15^c^	0.49 ± 0.06^e^
Control infected		4.16 ± 0.08^e^	2.51 ± 0.01 ^e^	0.26 ± 0.21^f^
Control healthy		6.17 ± 0.42^c^	4.11 ± 0.11^c^	0.22 ± 0.07^f^
LSD at 0.05		0.344	0.32	0.184

D - Metabolic indicators

To study the guidelines of the resistance in the infected tomato plants, the contents of protein, carbohydrates and free proline have been measured (Table 4). The data has shown that the control infected plants appeared a sharp decline in the contents of both protein and carbohydrates compared to healthy control. On the contrary, data showed that free proline significantly improved in infected plants as compared with healthy control plants. Interestingly the use of *Trichoderma* and SA either individual or combination through two modes (soil and foliar) enhanced the total soluble sugars and total soluble proteins contents in *Fusarium*-infected plants over infected plants only. The maximum recorded increase was observed in soluble sugars and soluble proteins contents was observed in (Tri + SA through foliar and SA through soil), followed by Tri + SA through soil, respectively. However, the contents of free proline incremented in infected plants and the highest values were noticed in plants treated with (Tri + SA, Tri and SA) and under infection through foliar, then followed by (Tri, Tri + SA and SA) through soil, respectively (see Table 5).

**Table 4:** Effect of Fusarium and (soil & foliar) application of *Trichoderma* and Salicylic acid and their interactions on the content of osmolytes (soluble sugars, soluble proteins and proline) of tomato plants.

Treatments	Method of application	Total carbohydrate	Total protein	Total proline
Tri	through soil	6.78 ± 0.26^c^	10.86 ± 0.42^c^	2.60 ± 0.01 ^bc^
SA		8.19 ± 0.03 ^a^	13.11 ± 0.04^b^	2.18 ± 0.11 ^e^
Tri + SA		8.03 ± 0.55^b^	12.85 ± 0.88^b^	2.45 ± 0.01 ^d^
Tri	through Foliar	6.13 ± 0.16 ^d^	9.29 ± 0.05^de^	2.81 ± 0.11^b^
SA		5.80 ± 0.03 ^de^	9.81 ± 0.25 ^d^	2.67 ± 0.01 ^bc^
Tri + SA		8.76 ± 0.33 ^a^	14.02 ± 0.53 ^a^	3.62 ± 0.13 ^a^
Control infected		5.41 ± 0.11^e^	8.66 ± 0.18 ^e^	1.47 ± 0.03^f^
Control healthy		9.03 ± 0.04 ^a^	14.45 ± 0.06 ^a^	1.25 ± 0.004 ^g^
LSD at 0.05		0.447	0.71	0.149

**Table 5:** **Effect of *Fusarium* and (soil & foliar) application of *Trichoderma* and Salicylic acid and their interactions on the content of osmolytes (**Polyphenol oxidase **and** Peroxidase **of tomato plants.**

Treatments	Method of application	Polyphenol oxidase	Peroxidase
Tri	through soil	0.68 ± 0.004 ^a^	0.55 ± 0.014 ^d^
SA		0.72 ± 0.01 ^de^	0.52 ± 0.003 ^de^
Tri + SA		1.03 ± 0.03 ^a^	0.79 ± 0.030^a^
Tri	through Foliar	0.80 ± 0.03^c^	0.61 ± 0.024^c^
SA		0.94 ± 0.06^b^	0.73 ± 0.05^b^
Tri + SA		1.06 ± 0.005 ^a^	0.82 ± 0.0039^a^
Control infected		0.95 ± 0.003^b^	0.68 ± 0.004 ^a^
Control healthy		0.63 ± 0.01^a^	0.72 ± 0.01 ^de^
LSD at 0.05		0.71	0.149

### 4.3 3- oxidative stress

**Fig. 3:**
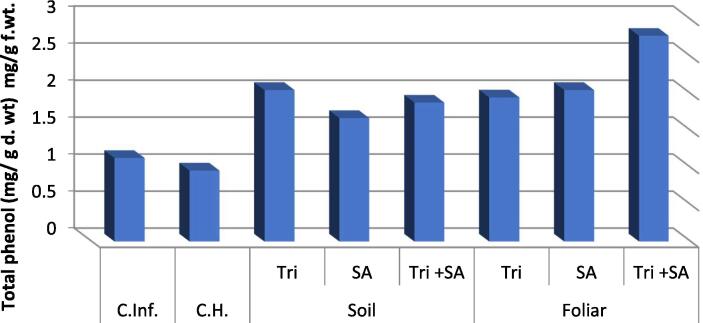
Effect of Fusarium and (soil & foliar) application of *Trichoderma* and Salicylic acid and their interactions on the content of Total phenols in tomato plants.

**Fig. 4:**
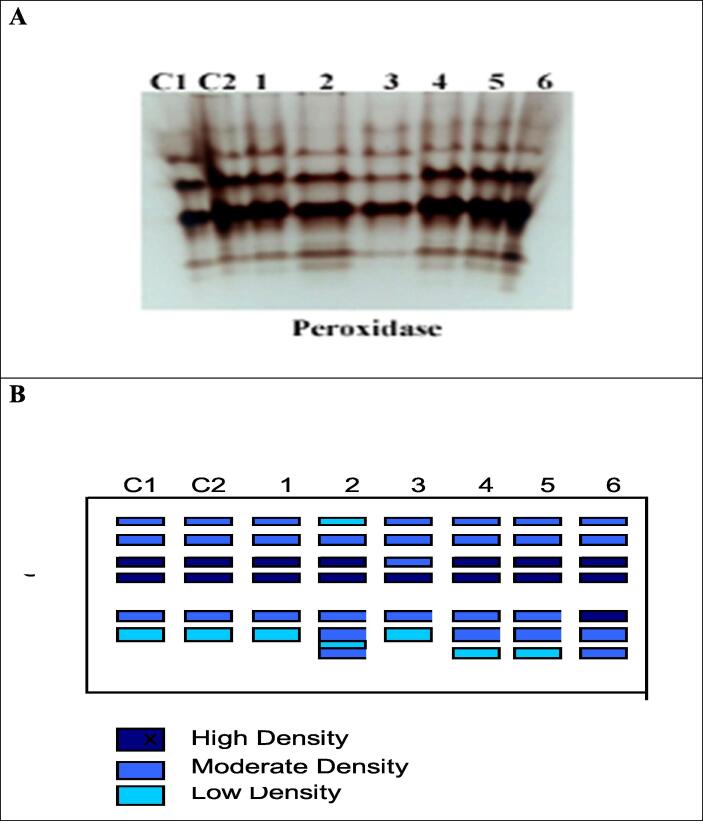
Effect of *Fusarium* and (soil & foliar) application of *Trichoderma* and Salicylic acid and their interactions on (A) Peroxidase isozyme and (B) Ideogram analysis of peroxidase isozyme of tomato plants.

It is apparent from Fig. 3 that plants treated with *Fusarium* showed significant increase in total phenol contents of by 15.04 % versus uninfected control (Fig. 4). Nonetheless, Tri + SA, Tri and SA foliar application was the best treatments and caused an evident rise in the phenol content. And came next, the soil application of Tri + SA, SA, and Tri showed a marked increment in the phenol content.

For antioxidant enzyme activities it is apparent from Figs. - and -- that there is a marked boost in the activity of POD and PPO under the *F. oxysporum* and/or *Trichoderma* and SA either soil or foliar application. Moreover, highly significant increases and maximum values for POD and PPO were observed due to application of Tri + SA on *F. oxysporum* infected plants through (foliar and soil) mode, then followed by SA (foliar), Tri (foliar), SA (soil) and Tri (soil) respectively in comparison to control infected plants. There were marked statistically significant increases (Fig. 3).

**Fig. 5:**
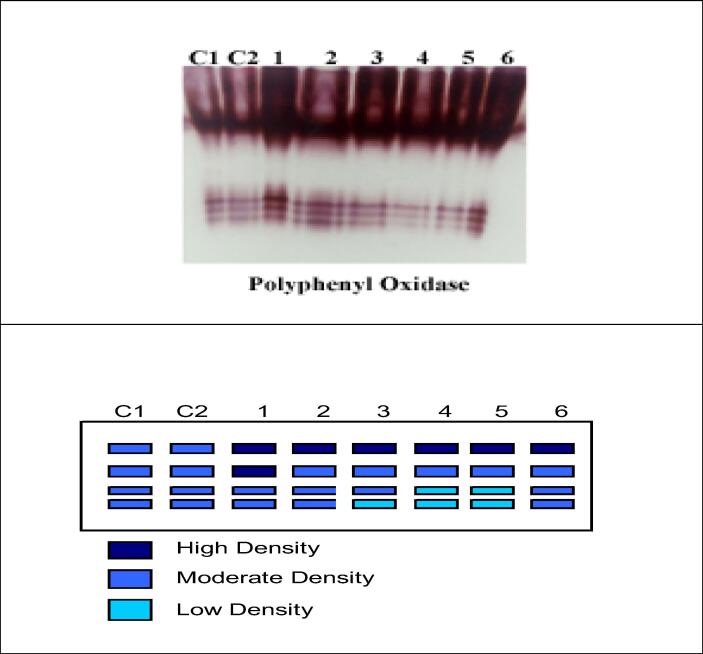
Effect of Fusarium and (soil & foliar) application of *Trichoderma* and Salicylic acid and their interactions on (A) Polyphenol oxidase isozyme and (B) Ideogram analysis of peroxidase isozyme of tomato plants.

E – Isozymes:

Foliar application of Tri + SA highly over-expressed the isozymes of POD that showed distinct 7 bands including 4 moderate at Rf (0.13, 0.29, 0.82 and 0.92) and 3 high dense band at Rf (0.36, 0.44 and 0.76), followed by Tri (foliar), SA (foliar and soil) treatments that showed the same 7 bands, 2 of them were highly dense at Rf (0.36 and 0.44) and 4 were moderate at Rf (0.189, 0.246, 0.861 and 0.861) and 1 was low at Rf (0.92) and came next (Tri and Tri + SA) through soil that gave 6 bands Fig. 4, Fig. 5 and Table 6.

**Table 6:** Isomers of peroxidase enzymes (+/−) and their Retention factor (Rf) in response to *Fusarium.*

**Perosidase groups**	**Relative Mobility**	**C1**	**C2**	**Soil**			**Foliar**		
				Tri	**SA**	**Tri + SA**	Tri	**SA**	**Tri + SA**
**Px 1**	0.1	1^+^	1^+^	1^+^	1^-^	1^+^	1^+^	1^+^	1^+^
**Px2**	0.2	1^+^	1^+^	1^+^	1^+^	1^+^	1^+^	1^+^	1^+^
**Px3**	0.3	1^++^	1^++^	1^++^	1^++^	1^+^	1^++^	1^++^	1^++^
**Px4**	0.4	1^++^	1^++^	1^++^	1^++^	1^++^	1^++^	1^++^	1^++^
**Px5**	0.7	1^+^	1^+^	1^+^	1^+^	1^+^	1^+^	1^+^	1^++^
**Px6**	0.8	1^-^	1^-^	1^-^	1^+^	1^-^	1^+^	1^+^	1^+^
**Px7**	0.9	0	0	0	1^-^	0	1^-^	1^-^	1^+^

++ High density Band + Moderate density Band – Low density Band 1 Present Band 0 Absent Band.

The isozyme of PPO contained 4 PPO isozymes in Fig. 4 and Table 7. Foliar application of (Tri) recorded highly over-expressed PPO that recorded 4 bands including 2 moderates at Rf (0.2 and 0.5) and 2 highly dense at Rf (0.6 and 0.7), followed by Tri + SA (foliar) and SA (soil) that gave 4 bands; 3 of them were moderate at (0.5, 0.6 and 0.7), and 1 was high dense at Rf (0.2). and came next, SA (foliar and soil) treatments that showed the same 7 bands, 2 of them were highly dense at Rf (0.36 and 0.44) and 4 were moderate at Rf (0.189, 0.246, 0.861 and 0.861) and finally 1 was low at Rf (0.92).

**Table 7:** Isomers of Polyphenyl Oxidase enzymes (+/−) and their Retention factor (Rf) in response to *Fusarium.*

**Polyphenyl Oxidase groups**	**Relative** **Mobility**	**C1**	**C2**	**Soil**			**Foliar**		
				Tri	**SA**	Tri	**SA**	Tri	**SA**
**PPO1**	0.2	1^+^	1^+^	1^++^	1^++^	1^++^	1^++^	1^++^	1^++^
**PPO2**	0.5	1^+^	1^+^	1^++^	1^+^	1^+^	1^+^	1^+^	1^+^
**PPO3**	0.6	1^+^	1^+^	1^+^	1^+^	1^+^	1^-^	1^-^	1^+^
**PPO4**	0.7	1^+^	1^+^	1^+^	1^+^	1^-^	1^-^	1^-^	1^+^

## 5 Discussion

*Fusarium* wilt disease is respected to be one of the mainly vital constraints across the globe. The developing information of abiotic pressures makes it critical to see other options that are found to be used in an easy way and possible to affect the destructive impacts of *Fusarium* wilt. The potentiality of myriads of controlling practices has been developed to minimize the deleterious impacts by either eradication of the pathogen or improve plant resistance (Heydari and Pessarakli, 2010, Newton et al., 2010, Ratnadass et al., 2012). In this context, the utility of novel and emerging mitigating tools could impart resistance of plant species under biotic stress. It is well known fact that in plants pathogenic infection resistance can be boosted via the biotic or abiotic inducers applied exogenously. It is more preferred to use vital and natural environmentally friendly elicitors for the fungus protection in crop plant rather than altering the whole micro flora of soil and enhanced resistance of plant diseases in order to preserve public health and the environment to suppress wide range of plant pathogens including *Fusarium* that caused wilt problems (Chaube et al., 2004, Latz et al., 2018, Pascale et al., 2020). This antifusarial activity explained by mycoparasitism way through disruption of fungal cell production of cell wall-lysing enzymes including chitinase, glucanase and protease wall as well as alternation of cytoplasmic components (Fig. 2) that is in harmony with (Bates et al., 1973, Dai et al., 1993, Irigoyen et al., 1992, Kashyap et al., 1980). Inhibition of *Fusarium* growth by *Trichoderma* through reduce and deformation of mycelium in dual culture method (Fig. 2) as competition mechanism proved by (Heydari and Pessarakli, 2010, Lavid et al., 2001). Another vital direct mechanism explained antifusarial activity by producing hook or knob-like structure and direct attack *Fusarium* (Newton et al., 2010). On another hand, results of TEM observation proved that the fungal cell components as well as morphology altered and damaged after SA treatment for more, destroyed the integrity of fungal plasma membrane, mitochondrial membrane that resulted to mitochondrial dysfunction and leakage of the fungal cytoplasmic contents and finally caused fungal cell death completely (Latz et al., 2018). The disease index in plants is the first guide against the pathogen to govern the occurrence of resistance. In this study there is clear evidence of resistance when applying *T. harzianum* and SA through two modes (foliar and soil) that results in the reduction of the disease percentage and severity and caused protection against infection. In the current study, both foliar and root applied *Trichoderma* and SA either (individual or combination) caused alleviative impacts to various growth traits. The growth traits were significantly boosted under *Trichoderma* and SA application either (individual or combination) under *F. oxysporum* infection. *F. oxysporum* resulted in the decline of studied plant growth parameters which is in accordance with the results of (Aldinary et al., 2021, Emamverdian et al., 2020, Kumar et al., 2005). This harmful effect in vegetative growth attributes due to *F. oxysporum* can be explained by accumulation of free ROS in cells and disturbances in enzymatic activity and photosynthesis process (Sharma et al., 2019). Photosynthesis plays a main anabolic role of plants, allowing plants to convert solar energy into biochemical energy which is successively used in all complex cell actions, and it is highly impacted by infection caused by various infections (Botero et al., 2018). In the recent study, *F. oxysporum* triggered a significant decrement in chlorophyll pigment contents, subsequent in a complete growth destroyed. These pigments were negatively affected by *Trichoderma* and SA either (individual or combination), this result became one of the obvious indications of treatment efficiency that can be discussed by *Trichoderma* ability to improve the soil and growth in the plant and supply plant with the nutrients (N, P, K) necessary to carry out the vital processes. It is clear from the present results that total phenol and proline content improved in infected plants and the greatest trend for total phenol content was noted in infected plants treated under *Trichoderma* and SA application, then followed by *Trichoderma* and SA, in comparison to control plants. The accumulation of phenolic compounds and proline in plant cells is evidence of the limitation of pathogen development, because these compounds are toxic to the pathogen and the plants use them as biochemical weapons for defense. Also, phenolic compounds may inhibit pathogen disease by enhancing the structural defense (Beckman and Roberts, 1995). In the current experiment, SA alone or with *Trichoderma* improved total phenols and free proline contents significantly in *F. oxysporum* treated plants. So, stimulation of total phenol content with SA and *Trichoderma* could exert a principal function in imparting resistance against *F. oxysporum*. These effects approve the accepted theory, that when infection happens to the plant cells, a change is triggered that shifts the normal primary metabolism into the secondary defense pathways, that results in the stimulation of myriads of genes encoding for defense enzymes (Farrag et al., 2017, Tarkowski et al., 2020). Such enrichment in the activities of antioxidants have been stated by others as well (Alhaithloul et al., 2020, Elkelish et al., 2020, Zaheer et al., 2020). It was noteworthy to see that observe that POD and PPO activities were found to be improved significantly due to application *Trichoderma* and SA either (individual or combination). SA is a main phenylpropanoid acid that stimulates resistance of plants against various pathogen stresses (Ojha and Chatterjee, 2012). Both modes of application of *Trichoderma* and SA either (individual or combination) showed significant more beneficial effects. The POD catalyses the H_2_O_2_ elimination (Das and Roychoudhury, 2014). Besides this in the present study, *Trichoderma* caused the highest phenolic accumulation which was in direct relationship with boosted activity of PPO. Thus, in final, the present addendum shows that stimulating the plant’s own resistance system by using antifusarial eco-friendly agents (*Trichoderma* and SA) can be an emerging approach in controlling various plant diseases.

## 6 Conclusion

The results of the experiment showed severe effects, including a decrease in morphological characteristics and metabolic processes, which led to oxidative brust in cells. It is concluded that using treatments *Trichoderma* and SA either (individual or combination) by foliar spray or soil treatment, which led to enhancement in immune responses and stimulation of substances responsible for defence within infected plants, thus reducing disease severity and increasing protection from disease. Therefore, the study recommends more studies on the use of these treatments in strengthening plant immunity and revealing the genes responsible for resistance to try to stay away from the use of harmful pesticides however; additional approaches should be employed to unravel actual underlying mechanisms.

## Declaration of Interests

The authors declare that there are no conflicts of interest related to this article.
